# More than the ‘master regulator of cytosolic proteostasis’: new roles for HSF1 in promoting tissue health

**DOI:** 10.1098/rstb.2025.0270

**Published:** 2026-10-01

**Authors:** John Labbadia

**Affiliations:** Institute of Healthy Ageing, Department of Genetics, Evolution and Environment, University College London, London WC1E 6BT, UK

**Keywords:** HSF1, proteostasis, stress responses, organelle homeostasis, healthy ageing

## Abstract

Maintaining protein homeostasis (proteostasis) is crucial for long-term tissue health. This requires the action of stress response pathways and protein quality control mechanisms that act within or across different sub-cellular compartments to preserve proteome integrity. Within the cytosol/nucleus, the loss of proteostasis induces a transcriptional programme known as the heat-shock response (HSR) through activation of heat-shock factor 1 (HSF1). The HSR rapidly elevates levels of molecular chaperones, co-chaperones and protein degradation factors that restore proteostasis in the cytosol/nucleus. As a result, the ability of HSF1 to promote tissue health has long been attributed to its capacity to safeguard the cytosolic/nucleosolic proteome. However, over the past 15 years, it has become apparent that HSF1 activity is also intimately coupled with the biogenesis and maintenance of other organelles, including mitochondria, peroxisomes, the endoplasmic reticulum, lysosomes and chloroplasts. This suggests that HSF1 promotes tissue health in plants and animals through mechanisms beyond the maintenance of cytosolic/nucleosolic proteostasis. In this opinion piece, I will discuss advances in our understanding of the interplay between HSF1 and organelle homeostasis and make the case that the existing model for the relationship between HSF1 and tissue health should be expanded to encompass these additional roles.

This article is part of the Theo Murphy meeting issue ‘ProteostaSys: a systems view of proteostasis’.

## 1. Heat-shock factor 1 and organismal health

Heat-shock factor 1 (HSF1) was first identified as the master regulator of the heat-shock response (HSR) in *Drosophila* 40 years ago [1,2]. Since this discovery, HSF1 has also been shown to be essential for normal development, growth, stem cell function and reproduction in worms, flies and mice [3–7], suggesting that HSF1 has evolved to not only promote viability in the face of acute toxic insults but also to allow cells to increase protein folding capacity and maintain proteostasis during periods of rapid cell growth, division and differentiation [8].

HSF1 is highly conserved, with invertebrates (worms, flies) possessing a single HSF [9]. By contrast, plants and mammals possess multiple HSFs that cooperate to regulate gene expression (reviewed comprehensively elsewhere [9–13]). In particular, mammalian HSF1 can form heterocomplexes with HSF2 to modulate the basal and stress-induced expression of proteostasis, metabolism and developmental genes within a variety of cellular contexts, including differentiation, alcohol exposure, proteasome inhibition, spermatogenesis and cancer formation [14–21]. Given its broad conservation across organisms and its established role as the master regulator of proteostasis across species, in this review, I focus exclusively on recent developments concerning mammalian HSF1 and its orthologues. As the nomenclature for HSF1 differs across species (e.g. Hsf1, HSF1, HSF-1, HSF) [9], I will use ‘HSF1’ throughout and refer to the relevant species being explored in the text.

The regulation of HSF1 is a tightly controlled process that is dependent on post-translational modifications, interactions with co-factors and the chromatin landscape (reviewed comprehensively elsewhere [9,10]). However, at its core, the activation/repression cycle of HSF1 revolves around its association with the molecular chaperone, heat-shock protein 70 (HSP70) [22,23]. In response to the appearance of misfolded proteins in the cytosol/nucleus, HSP70 is titrated away from HSF1, allowing HSF1 monomers to form functional homotrimers within the nucleus [22,23]. HSF1 trimers possess DNA binding competency and bind to heat-shock elements (HSEs) located within the promoters of genes encoding HSP70, HSP40, HSP90 and small HSP family members, as well as nucleotide exchange factors, proteasomal subunits and ubiquitylation enzymes, thereby promoting the expression of these genes [24,25]. This response (commonly referred to as the HSR) enhances protein folding capacity in the cytosol/nucleus, allowing cells to more efficiently sequester, refold or degrade misfolded proteins and restore proteostasis [26]. Once proteostasis has been restored, HSP70 is free to bind to HSF1 trimers, thereby promoting their disassembly back into monomers [22,23].

Consistent with its role in driving the HSR, it is well established that HSF1 is required for cells to respond to, and neutralize, the toxic effects of protein misfolding, mislocalization and/or aggregation caused by exposure to elevated temperatures, infection, environmental toxins or genomic mutations [26]. Furthermore, reduced HSF1 activity is associated with increased susceptibility to protein aggregation and shortened lifespan in *Caenorhabditis elegans* [27,28], whereas increased HSF1 activity promotes longevity and suppresses the onset and progression of age-associated protein conformational diseases in worms, flies and mice [26]. In addition, HSF1 activity declines in the tissues of aged worms [29,30] and rodents [31–33], and in human cells undergoing replicative senescence [34,35]. This has been attributed to reduced DNA binding activity as a consequence of changes in HSF1 protein levels and post-translational modifications, reduced nuclear translocation and altered chromatin accessibility [29–35]. Together, these observations suggest that a reduction in HSF1 activity with age contributes to age-associated tissue dysfunction. However, the relationship between HSF1 and organismal health is complex, with increased HSF1 activity also promoting the survival of cancer cells and tumorigenesis [36].

While HSF1 is intimately coupled with health, the mechanisms that operate downstream of HSF1 to promote proper tissue function remain poorly understood. For over 30 years, it has been assumed that the beneficial impact of HSF1 activation on cells and tissues stems exclusively from protection of cytosolic/nuclear proteins. However, over the past 10 years, HSF1 has emerged as an integral contributor to the maintenance of mitochondria and peroxisomes, the endoplasmic reticulum (ER) and Golgi, the endolysosomal system and chloroplasts. In this review, I provide an overview of these findings and argue for a move away from the traditional view of HSF1 as ‘the master regulator of cytosolic/nuclear proteostasis’ and towards an integrated view of HSF1 as ‘the master regulator of cellular proteostasis’.

## 2. HSF1 and cytosolic/nuclear protein homeostasis

The canonical target genes of HSF1 encode cytosolic/nuclear members of the HSP70, HSP40, HSP90 and small heat shock protein (sHSP) molecular chaperone families. In yeast, the co-expression of just two cytosolic/nuclear molecular chaperones, HSP70/SSA2 and HSP90/HSC82, is sufficient to restore growth under basal conditions when HSF1 activity is impaired [37]. However, these cells are slow-growing, suggesting HSF1 may also act through other mechanisms to promote full cellular robustness [37]. Alongside these genes, the HSR is also characterized by the upregulation of nucleotide exchange factors, such as BAG3 and HSP110, and components of the ubiquitylation machinery and proteasome [24,25]. The mechanisms by which these factors promote the folding/re-folding, trafficking or degradation of proteins in the cytosol and nucleus, and the disassembly of protein aggregates, have been comprehensively described elsewhere [38–44]. These chaperones protect cells by interacting with, and helping to maintain, hundreds of cytosolic/nuclear proteins involved in mRNA processing, signalling, vesicle trafficking, organelle import, translation, metabolism and chromatin organization [45–47].

A comparison of the HSR and developmental programmes regulated by HSF1 in *C. elegans* has revealed that the transcriptional programme driven by HSF1 during development includes core molecular chaperones, such as HSP70, HSP90, HSP40 and subunits of the T-complex protein ring (TRiC), but is distinct from the HSR3. Likewise, an examination of HSF1 activity in *ex vivo* cultured mouse and human haematopoietic stem cells (HSCs) revealed that maintenance of proteostasis by HSF1 occurs through upregulation of non-chaperone proteins in response to a rapid elevation in protein synthesis [5]. Finally, larval lethality in *Drosophila* HSF1 mutants was not associated with reduced expression of canonical HSR genes [4], while an examination of stress-inducible genes in yeast revealed that SSA1/HSP70 contains HSEs and is strongly induced by heat-shock but repressed by other stresses [48]. Together, these observations suggest that the mechanisms by which HSF1 safeguards cytosolic proteostasis and promotes proper cell and tissue function in the absence of environmental stress are dependent on life stage, tissue-type and form of proteome challenge encountered, and are not simply owing to activation of the HSR.

HSF1-regulated chaperones, most notably HSP70, are also incredibly important for ensuring that protein biogenesis proceeds as required and that any errors are rapidly dealt with by interacting with nascent polypeptide chains at the ribosome [49,50]. In addition, a screen for genes that activate HSF1 when knocked down in *Saccharomyces cerevisiae* revealed that translation stress leads to a robust, HSF1-dependent stress response that is sensed differently from the canonical HSR [51]. This response is triggered by impairment of Rqc1 and Ltn1 (an E3 ubiquitin ligase), which recruit Cdc48 to a ribosome quality control (RQC) complex that interacts with stalled polypeptides and directs them for degradation [51]. In addition, HSF1 has been shown to recruit GCN5 to promote the c-MYC-mediated regulation of genes involved in ribosome function and biogenesis [52].

Finally, increased HSF1 activity has also been reported to maintain cytoskeletal integrity in response to thermal stress and ageing [53,54]. Expression of an HSF1 variant that was unable to promote chaperone expression in response to stress stabilized actin filaments by promoting the expression of the calcium binding protein, PAT-10/CALML6 [53]. Furthermore, HSF1 activity was found to be crucial for the maintenance of cytoskeletal integrity with age in various tissues of *C. elegans* [54]. Together, these findings make it abundantly clear that HSF1 has a highly conserved, primary role in safeguarding the cytosolic/nuclear proteome against environmental stress and age-related changes. However, given the inter-dependence of various organelles for their biogenesis and maintenance, is the cytosol/nucleus the only sub-cellular location that benefits from HSF1 activity?

## 3. HSF1 promotes mitochondrial homeostasis

The biogenesis and maintenance of mitochondrial homeostasis are heavily dependent on cytosolic proteostasis networks [55,56]. The vast majority of mitochondrial proteins are synthesized in the cytosol and trafficked to the outer mitochondrial membrane before being imported via dedicated import factors/pathways. These precursor proteins are then processed and either inserted into the inner membrane, shuttled to the intermembrane space or pulled into the matrix [55,56]. Cytosolic HSP70 and HSP90 are essential for this process to proceed efficiently, as they transport mitochondrial precursor proteins through the cytosol to the outer mitochondrial membrane import receptor, TOM70 [57]. Moreover, blocking mitochondrial protein import strongly activates an HSF1-dependent mitoprotein-induced stress response in yeast, presumably to protect the cytosol against the potentially toxic effects of orphaned mitochondrial precursor proteins [58,59].

As well as directly regulating the expression of cytosolic HSP70 and HSP90, HSF1 can also promote the expression of mitochondrial chaperones, such as HSP60 and HSP10 [60]. Recent findings in cancer cells have shown that HSF1 drives mitochondrial biogenesis by increasing steady-state levels of the inner mitochondrial membrane transporter, TIMM17A, through expression of HSP60 and HSP10 [60]. This facilitates the translocation of new proteins into the matrix, thereby remodelling the mitochondrial proteome to promote translation and energy production [60]. In addition to promoting chaperone expression, overexpression of HSF1 has also been shown to increase the expression of the outer mitochondrial membrane quality control factor, ubiquilin-1 [61] in *C. elegans* [62]. This results in reduced levels of yeast nuclear protein localization-4 (NPL-4), increased mitochondrial fusion and extended lifespan [62].

If mitochondrial function is compromised or protein folding capacity is exceeded, cells will activate a mitochondrial unfolded protein response (UPR^mt^) that elevates mitochondrial chaperones and proteases, thereby restoring mitochondrial proteostasis [63]. While first discovered in mammalian cells, the core principles behind the UPR^mt^ have been largely explored and explained in *C. elegans,* and have revealed that in worms, impaired mitochondrial protein import leads to relocation of the transcription factor ATFS-1 from mitochondria to the nucleus [63]. When in the nucleus, ATFS-1 cooperates with the transcription factor DVE-1/SATB1, which drives the UPR^mt^, leading to the upregulation of genes that encode mitochondrial chaperones, proteases, metabolic enzymes and electron transport chain components [63].

In addition to this core UPR^mt^, disrupting mitochondrial proteostasis by knocking down HSP-6—the mitochondrial HSP70—leads to a strong mitochondria-to-cytosolic stress response that is dependent on cooperation between ATFS-1, DVE-1 and HSF1 [64]. This response is dependent on reduced fatty acid beta-oxidation, reduced levels of ceramide and increased levels of cardiolipin, and acts to simultaneously preserve mitochondrial homeostasis and suppress cytosolic protein aggregation in worms and human fibroblasts [64]. Similarly, impairing respiration in *C. elegans* through RNAi knockdown of electron transport chain (ETC) components, or knockdown of the outer mitochondrial membrane insertase MTCH-1/MTCH2, also results in an HSF1-mediated cytosolic stress response that is distinct from the HSR [65–67]. Activation of HSF1 in response to reduced ETC activity occurs through dephosphorylation of HSF1 by the protein phosphatase PP2A complex [66], which leads to the preferential induction of small HSPs and HSP70 [66]. Interestingly, PP2A also promotes a more active form of HSF1 in human cancer cells through hypo-phosphorylation, suggesting that links between ETC activity, PP2A and the regulation of HSF1 are highly conserved from worms to humans [68].

In mammalian cells, it has emerged that HSF1 is required for full activation of the UPR^mt^ in mouse fibroblast cells exposed to the mitochondrial HSP90 inhibitor, G-TTP, the LON protease inhibitor, CDDO or the ETC complex I inhibitor, rotenone [69,70]. HSF1 activation by mitochondrial stress in mammalian cells is mediated by the accumulation of mitochondrial precursor proteins and the oxidation of DNAJA1 in the cytosol by mitochondrial reactive oxygen species [70]. This leads to the efficient titration of HSP70 away from HSF1 to mislocalized mitochondrial precursor proteins, thereby allowing for HSF1 trimerization and activation [70]. In addition, heat stress leads to mobilization of single-stranded DNA-binding protein, mitochondrial (SSBP1) away from mitochondrial DNA and into the nucleus via the mitochondrial transition permeability pore, ANT-VDAC1 [71]. This potentiates HSF1 activity through the recruitment of the chromatin remodelling factors BRG1 and improves survival of cells exposed to heat stress [71].

HSF1 activation in response to mitochondrial stress is consistently associated with elevated levels of small HSPs. In mammalian cells, small HSPs have been shown to migrate from the cytosol to the mitochondrial intermembrane space, where they act as chaperones to support efficient protein folding and complex assembly by suppressing protein aggregation [72]. Preventing this process leads to mitochondrial swelling and impaired respiration [72], indicating that HSF1 promotes mitochondrial health and function not only by promoting levels of chaperones dedicated to the cytosol or mitochondrial matrix but also by increasing levels of ATP-independent chaperones that can soak up misfolded proteins within both compartments.

## 4. HSF1 promotes peroxisome biogenesis and function

Peroxisomes are dynamic organelles that are formed through budding and fusion of vesicles from the ER, and that can generate acetyl-coA for mitochondria through fatty acid beta-oxidation [73]. This generates hydrogen peroxide, which is converted into water within peroxisomes through catalase [73]. As such, peroxisomes have a central role in lipid and redox homeostasis within cells [73]. Recent work in yeast has shown that when the major ER HSP70, Kar2/BiP, is impaired, cells promote peroxisome biogenesis through activation of HSF1 and inhibition of TOR1 [74]. In addition, expression of a dominant negative version of HSF1 suppresses the folding of peroxisome-targeted luciferase post heat-shock in human cells [75]. Moreover, full induction of *hsp-16* genes following heat-shock is impaired in *C. elegans* catalase defective *clt-2* mutants [76]. Together, these studies reveal an interplay between peroxisome biogenesis and function, and HSF1 activity.

HSF1 has also been shown to cooperate with peroxisome proliferator-activated receptor gamma coactivator-1α (PGC1α), a transcriptional co-activator that drives mitochondrial and peroxisome biogenesis [77–79]. In response to hyperthermia, PGC1a associates with HSF1 at gene promoters to promote the expression of molecular chaperones [77]. In addition, activation of HSF1 in mouse fat and muscle tissue promotes a PGC1a-dependent metabolic programme that protects against the damaging effects of a high fat diet [78]. Conversely, upon exposure to fasting, PGC1a associates with HSF1 at gene promoters in mouse livers and primary hepatocytes to repress the expression of molecular chaperones [79]. These findings reveal that HSF1 activity is intimately coupled with metabolic responses through coupling with PGC1a and that this leads to stress and tissue-specific responses to environmental challenges.

## 5. HSF1 protects the secretory pathway

The secretory pathway describes the flow of newly synthesized proteins from the ER lumen to cellular release via COPII vesicles and the Golgi complex [80]. Cells protect against the disruption of protein homeostasis within these compartments through the activation of numerous protein quality control mechanisms, including the ER unfolded protein response (UPR^ER^) and ER-associated degradation (ERAD), both of which have been comprehensively reviewed elsewhere [81,82].

Interestingly, a *C. elegans* genome-wide RNAi screen for negative regulators of HSF-1 activity found that the knockdown of COPI proteins (F38E11.5 and T14G10.5), ER HSP70 chaperones (hsp-3 and T24H7.2) and signal recognition particle subunits (F38A1.8, R186.3, F55C5.8, F08D12.1 and F25G6.8) increased HSF-1 activity in the intestine, a major secretory tissue in worms [83]. This suggests that cells respond to the disruption of ER homeostasis by activating HSF1. In addition, a recent study suggests that HSF1 is required for full induction of UPR^ER^ genes in response to tunicamycin treatment in *C. elegans* and human cell lines [84].

Attempts to identify novel HSF1 interaction partners in *C. elegans* revealed that COPII-associated proteins SAR-1, NPP-20, SEC-23 and SEC-24 are all recovered when HSF1 is immunoprecipitated from *C. elegans* tissues [85]. In addition, knocking down *sec-23* through RNA interference (RNAi) resulted in increased nuclear HSF-1, elevated levels of HSP70 (*hsp-70* and *F44E5.4*) and small HSP (*hsp-16.2*) mRNA, and resistance to polyglutamine aggregation and heat stress [85]. Using GFP-tagged VIT-2 and DAF-28 as model secretory proteins, it was shown that knockdown of SAR-1 blocked protein secretion, which was exacerbated, leading to protein aggregation, when HSF-1 was also knocked down [85]. Moreover, SEC-23 was also shown to interact with HSF1 in human cells and to colocalize with HSF1 at the ER in mouse fibroblasts. Depletion of SAR1 in mouse fibroblasts by siRNA disrupts this interaction. These observations suggest that HSF1 activation simultaneously protects the cytosol while also helping cells and tissues to maintain protein secretion [85].

Studies of how HSF1 influences ER homeostasis in yeast have shown that the expression of a constitutively active form of HSF1 rescues export defects in *ire1* deletion mutants overexpressing CPY* [86]. These effects are associated with elevated levels of the COPII vesicle cargo receptors, ERV29 and EMP24, and the ER HSP70, Kar2/BiP [86]. This also suppresses death of *ire1*-deficient cells caused by the glycosylation inhibitor, tunicamycin. Moreover, in *ire1* deletion yeast but not wild-type cells, ER stress caused by CPY* overexpression or tunicamycin treatment leads to increased HSF1 activity [86]. It is probable that the coupling of HSF1 to ER protein import and secretion acts to ensure that any ER proteins orphaned in the cytosol are efficiently sequestered or degraded, thereby preventing protein aggregation. However, it is possible that HSF1 protects components of the secretory pathway directly. Under conditions of ER stress, activation of HSF1 by brief heat-shock preconditions cells against subsequent exposure to elevated temperatures [87]. This is mediated by the induction of small HSPs, which are found to localize to the Golgi [87]. Embryonic stem cell-derived glutamatergic neurons and primary mouse cortical and striatal neurons are also protected from heat-induced death by overexpression of the sHSPα-crystallin, which was found to localize to the Golgi [87].

Finally, HSF1 activity has been shown to be coupled with the ER membrane kinases, IRE1 and PERK, which act to signal protein folding stress within the ER lumen to the nucleus in order to activate different branches of the UPR^ER^. Exposure of yeast to ER stress through treatment with tunicamycin or dithiothreitol has been found to increase HSF1 activity (as measured by a B-Gal reporter system) in a manner that was dependent on *ire1* and *hac1* [88]. In addition, in mouse brain tissue, HSF1 deficiency (HSF-1+/−) leads to activation of PERK and increased aggregation of hyperphosphorylated tau protein with age [89]. Together, these studies indicate that in addition to maintaining cytosolic proteostasis, HSF1 also promotes transcriptional programmes that simultaneously support function throughout the secretory system, either at the ER membrane or within the Golgi complex.

## 6. HSF1 regulates lysosomal activity

Lysosomes are membrane-bound proteolytic organelles that degrade material imported into cells via endocytosis and endosomes, and they destroy organelles and proteins sequestered within autophagosomes by various forms of autophagy [90]. In addition, individual proteins can be directed to lysosomes for degradation through chaperone-mediated autophagy [90].

Given the central role of lysosomes in maintaining protein homeostasis, it is unsurprising that HSF1 activity has been linked to lysosomal biogenesis and increased protein degradation. Overexpression of HSF1 in *C. elegans* leads to elevated expression of genes that encode vacuolar ATPases and lysosome trafficking factors [91] and is associated with enhanced autophagy within the hypodermis, neurons, muscles and intestinal tissues [91]. This is also associated with enhanced stress resistance, protection from protein aggregation and increased lifespan [91]. Consistent with this, heat stress also leads to increased autophagy in an HSF1-dependent manner [91]. Conversely, in cancer cells, human kidney cells and mouse fibroblasts, HSF1 has been shown to cooperate with the transcription factor c-MYC to repress the expression of lysosomal and autophagy genes [52]. These findings suggest that HSF1 simultaneously enhances the expression of molecular chaperones and protein clearance pathways via the autophagy/lysosome system. This may expedite the clearance of misfolded proteins that cannot be refolded by molecular chaperones and/or ensure that any protein aggregates that are formed are rapidly removed.

## 7. HSF1 promotes the chloroplast unfolded protein response

Plants have more than 20 different HSF family members that are split into three classes, A, B and C. These have been most intensively studied in *Arabidopsis*, tomato and rice, with HSFA family members most closely resembling mammalian HSF1 [12].

Like mitochondria, chloroplast biogenesis and maintenance rely on the synthesis of preproteins in the cytosol, subsequent transport of preproteins to the chloroplast outer membrane, and import via dedicated receptors, channel proteins and molecular ratchets. As with mitochondrial protein import, this process is dependent on cytosolic chaperones, such as HSP70 and HSP90 [92]. Also like mitochondria, the loss of protein homeostasis within chloroplasts owing to phototoxicity, temperature or chemical stress, triggers a chloroplast unfolded protein response (cpUPR) that elevates levels of chloroplast chaperones and proteases in order to maintain and/or restore chloroplast function. In *Arabidopsis*, inhibiting protein synthesis within chloroplasts through lincomycin treatment caused protein aggregation and resulted in a cpUPR characterized by elevated mRNA levels of HSFA2 and induction of the HSFA2 target chaperone genes *ClpB3*, *Hsp70.2* and *Hsp21* [93]. In addition, the chloroplast ribosomal protein, RPS1 has been shown to be essential for full induction of the cytosolic stress response by HSFA2. Loss of RPS1 blunted the HSFA2 response to protein folding stress, destabilized thylakoid membranes and made plants vulnerable to heat stress [94]. Finally, it has been well documented that plants are more resistant to heat stress during the day than at night. This phenomenon is linked to light-induced hydrogen peroxide generation by chloroplasts, which signals to HSFA1 and promotes HSP70 expression [95]. Together, these findings demonstrate that HSFs are crucial for chloroplast maintenance and reveal an intimate connection between chloroplast integrity and HSF1 activity.

## 8. Reframing HSF1 as the master regulator of cellular proteostasis

HSF1 has long been recognised as a stress responsive regulator of HSP genes and the master regulator of cytosolic/nuclear proteostasis. This view remains as true today as it did 30 years ago; however, in light of advances in our understanding of HSF1, I would argue that this tagline does HSF1 a disservice. A substantial body of evidence now makes it clear that the beneficial effects of HSF1 are far more widespread across the cell than originally recognised. As such, we should revise our thinking and recognise that HSF1 is the master regulator of proteostasis across multiple compartments of the cell.

But is this position deceptive? HSF1 activity is integrated into organelle-specific UPRs, import pathways and degradation pathways, but it is impossible to ignore the fact that it is the upregulation of cytosolic chaperones that often drives its beneficial effects on the biogenesis, function and maintenance of other organelles. Indeed, the primary role of HSF1 is still to protect the cytosol and nucleus, with beneficial effects on mitochondria or the ER arising from safeguarding of the cytosolic proteome. Studies showing direct effects of HSF1 on the expression of mitochondria or ER-specific chaperones suggest a cytosol/nuclear-independent role for HSF1; however, this is never uncoupled from a parallel increase in cytosolic HSP70 or small HSPs. Nevertheless, the interdependence of different compartments on one another for proper maintenance and function still positions HSF1 as the central cellular determinant of proteome health, even if protecting the cytosolic/nuclear proteome is its immediate objective.

Over the past 10 years, work from many groups has expanded our understanding of the interplay between HSF1, proteostasis and cell function. However, many important questions remain unanswered, such as: (i) why is HSF1 activated in response to some organellar insults but not others, (ii) how and why are HSF1-dependent transcriptional responses tailored to the type of organellar protein folding stress encountered, (iii) are different HSF1-dependent responses observed in all tissues, (iv) does the response of HSF1 to organellar stress change throughout life, and (v) which HSF1-directed transcriptional programmes promote the health of different tissues? Answering these questions will allow us to develop a fully integrated view of HSF1’s role in promoting cell and tissue function throughout life, thereby paving the way for the development of effective strategies that promote healthy ageing.

## Data Availability

This article has no additional data.

## Theme

One contribution of 20 to a Theo Murphy meeting issue ‘ProteostaSys: a systems view of proteostasis’.
